# The Neural Correlates of Religious and Nonreligious Belief

**DOI:** 10.1371/journal.pone.0007272

**Published:** 2009-10-01

**Authors:** Sam Harris, Jonas T. Kaplan, Ashley Curiel, Susan Y. Bookheimer, Marco Iacoboni, Mark S. Cohen

**Affiliations:** 1 UCLA Ahmanson-Lovelace Brain Mapping Center, David Geffen School of Medicine, University of California Los Angeles (UCLA), Los Angeles, California, United States of America; 2 Brain and Creativity Institute and Department of Psychology, University of Southern California (USC), Los Angeles, California, United States of America; 3 Department of Clinical Psychology, Pepperdine University, Los Angeles, California, United States of America; 4 Semel Institute for Neuroscience and Human Behavior, University of California Los Angeles (UCLA), Los Angeles, California, United States of America; 5 Center for Cognitive Neuroscience, University of California Los Angeles, Los Angeles (UCLA), Los Angeles, California, United States of America; 6 Departments of Psychiatry and Biobehavioral Sciences, University of California Los Angeles (UCLA), Los Angeles, California, United States of America; 7 The Brain Research Institute, University of California Los Angeles (UCLA), Los Angeles, California, United States of America; 8 Departments of Neurology, Radiological Sciences, Biomedical Engineering, and Biomedical Physics, University of California Los Angeles (UCLA), Los Angeles, California, United States of America; 9 Department of Psychology, University of California Los Angeles (UCLA), Los Angeles, California, United States of America; 10 The Reason Project, Santa Monica, California, United States of America; Indiana University, United States of America

## Abstract

**Background:**

While religious faith remains one of the most significant features of human life, little is known about its relationship to ordinary belief at the level of the brain. Nor is it known whether religious believers and nonbelievers differ in how they evaluate statements of fact. Our lab previously has used functional neuroimaging to study belief as a general mode of cognition [1], and others have looked specifically at religious belief [2]. However, no research has compared these two states of mind directly.

**Methodology/Principal Findings:**

We used functional magnetic resonance imaging (fMRI) to measure signal changes in the brains of thirty subjects—fifteen committed Christians and fifteen nonbelievers—as they evaluated the truth and falsity of religious and nonreligious propositions. For both groups, and in both categories of stimuli, belief (judgments of “true” vs judgments of “false”) was associated with greater signal in the ventromedial prefrontal cortex, an area important for self-representation [3], [4], [5], [6], emotional associations [7], reward [8], [9], [10], and goal-driven behavior [11]. This region showed greater signal whether subjects believed statements about God, the Virgin Birth, etc. or statements about ordinary facts. A comparison of both stimulus categories suggests that religious thinking is more associated with brain regions that govern emotion, self-representation, and cognitive conflict, while thinking about ordinary facts is more reliant upon memory retrieval networks.

**Conclusions/Significance:**

While religious and nonreligious thinking differentially engage broad regions of the frontal, parietal, and medial temporal lobes, the difference between belief and disbelief appears to be content-independent. Our study compares religious thinking with ordinary cognition and, as such, constitutes a step toward developing a neuropsychology of religion. However, these findings may also further our understanding of how the brain accepts statements of all kinds to be valid descriptions of the world.

## Introduction

Since the 19^th^ century, it has been widely assumed that the spread of industrialized society would spell the end of religion. Marx [12], Freud [13], [14], and Weber [15]—along with innumerable anthropologists, sociologists, historians, and psychologists influenced by their work—expected religious belief to wither in the light of modernity. It has not come to pass. Religion remains one of the most prominent features of human life in the 21^st^ century. While most developed societies have grown predominantly secular [16], with the curious exception of the United States, orthodox religion is in full bloom throughout the developing world. Indeed, humanity seems to becoming proportionally more religious, as the combination of material advancement and secularism is strongly correlated with decreased fertility [17]. When one considers the rise of Islamism throughout the Muslim world, the spread of Pentecostalism throughout Africa, and the anomalous piety of the United States, it becomes clear that religion will have geopolitical consequences well into the 21st century.

Given the importance of religion in human life, surprisingly little is known about its basis in the brain. The relevance of the brain's ventromedial dopaminergic systems to religious experience, belief and behavior is suggested by several lines of evidence, including the fact that a variety of clinical conditions related to dopaminergic dysfunction—mania, obsessive-compulsive disorder (OCD), schizophrenia, and temporal-lobe epilepsy—are regularly associated with hyperreligiosity [18]. The serotonergic system has also been implicated, as drugs known to modulate it—like LSD, psilocybin, mescaline, N,N-dimethyltryptamine (“DMT”), and 3,4-methylenedioxymethamphetamine (“ecstasy”)—seem especially potent drivers of religious/spiritual experience. In addition, 5-HT1A receptor densities have been inversely correlated with high scores on the “spiritual acceptance” subscale of the Temperament and Character Inventory [19].

There have been a number of neuroimaging and EEG studies done on religious practice and experience—primarily focusing on meditation [20], [21], [22], [23], [24] and prayer [25], [26], [27], [28], [29], [30]. The purpose of these studies has been to evoke spiritual/contemplative experiences in religious subjects and to compare these states of mind to a control condition. However, none of these studies were designed to isolate the variable of belief itself, or to determine whether religious belief differs from ordinary belief at the level of the brain.

As many have noted, religion cannot be reduced to a mere concatenation of religious beliefs. Every religion consists of rites, rituals, prayers, social institutions, holidays, etc., that serve a wide variety of purposes, explicit or otherwise [31], [32]. However, religious *belief*—that is, the acceptance of specific religious propositions as being true—is generally what renders these enterprises relevant, or even comprehensible. While there may be many Catholics, for instance, who value the ritual of the Mass without actually believing the doctrine of Transubstantiation, the primacy of the Mass within the Church still hinges on the fact that many Catholics do accept it as a metaphysical truth—a fact that can be directly attributed to specific, doctrinal claims that are still put forward by the Church. There is, of course, a distinction to be made between mere *profession* of such beliefs and actual *belief* [33]—a distinction that, while important, only makes sense in a world in which some people actually believe what they say they believe. There seems little reason to doubt that a significant percentage of human beings, likely a majority, falls into this latter category with respect one or another religious creed.

Our lab published the first neuroimaging study of belief as a general mode of cognition [1], and another group has looked specifically at religious conviction [2]. However, no research has compared these two states of mind directly. Here we show that while religious and nonreligious thinking differentially engage broad regions of the frontal, parietal, and medial temporal lobes—and, hence, appear quite distinct as modes of thought—the difference between belief and disbelief appears to be content-independent.

## Results

We used functional magnetic resonance imaging (fMRI) to measure signal changes in the brains of thirty subjects—fifteen committed Christians and fifteen nonbelievers—as they evaluated the truth and falsity of religious and nonreligious propositions. For each trial either a religious statement (*e.g.*, “Jesus Christ really performed the miracles attributed to him in the Bible”) or a nonreligious statement (*e.g.*, “Alexander the Great was a very famous military leader”) appeared, and participants pressed a button to indicate whether the statement was true or false. Our stimuli were designed to produce roughly equal numbers of believed and disbelieved trials in each category.

### Behavioral data

Response time data were submitted to a repeated-measures ANOVA with belief (true, false) and statement content (religious, nonreligious) as within-subject variables, and group (nonbeliever, Christian) as a between-subject variable. Response times were significantly longer for false (3.95 s) compared to true (3.70 s) responses (F (1,28) = 33.4, p<.001), and also significantly longer for religious (3.99 s) compared with nonreligious (3.66 s) stimuli (F (1,28) = 18, p<.001). The two-way interaction between belief and content type did not reach significance, but there was a three-way interaction between belief, content type, and group (F (1,28) = 6.06, p<.05). While both groups were quicker to respond “true” than “false” on both categories of stimuli, the effect of truth was especially pronounced for nonbelievers when responding to religious statements (see Supplementary Information: Table S1 and Figure S1).

### Belief compared with disbelief

**Figure 1:**
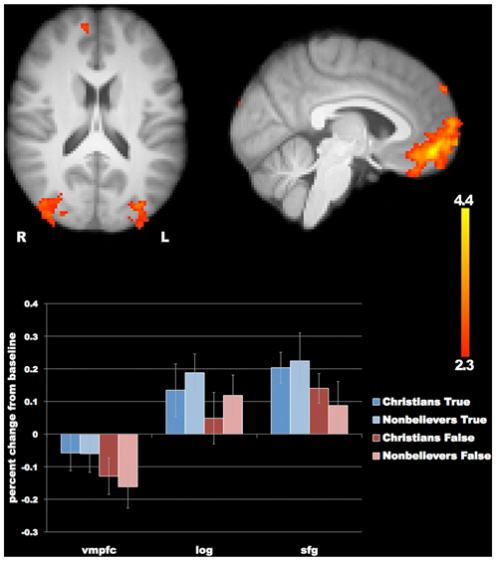
Belief minus disbelief (Both Categories; Both Groups). Greater signal for belief compared with disbelief appeared in the ventromedial prefrontal cortex, lateral occipital cortex, and superior frontal gyrus. The bottom panel shows percent signal change from baseline in each of the clusters (vmpc = ventromedial prefrontal cortex; log = lateral occipital gyrus; sfg = superior frontal gyrus). Error bars represent standard error of the mean.

For both groups, and in both categories of stimuli, belief was associated with greater blood-oxygen-level-dependent (BOLD) signal in the ventromedial prefrontal cortex (VMPFC, see Fig. 1, Table 1), an area important for self-representation [3], [4], [5], [6], emotional associations [7], reward [8], [9], [10], and goal-driven behavior [11]. This region showed greater signal whether subjects believed statements about God, the Virgin Birth, etc. or statements about ordinary facts. We also saw greater signal in the left superior frontal gyrus and in both lateral occipital cortices for this contrast.

**Table 1:** Belief minus disbelief.

Brain region	hemi	x	y	z	Peak Z score
Ventromedial prefrontal	L	−4	50	−12	4.42
Superior frontal gyrus	L	−22	32	50	4.06
Lateral occipital cortex	L	−30	−82	16	3.19
	R	44	−88	12	3.61

The differences in VMPFC signal were due to a greater relative decrease in activation from baseline for the disbelief condition. Our finding of greater signal in VMPFC for belief compared to disbelief was significant in both Christians and nonbelievers for both religious and nonreligious stimuli, supporting a role for this brain region in the acceptance of truth-claims across content domains. A direct comparison of *belief minus disbelief* in Christians and nonbelievers did not show any significant group differences for nonreligious stimuli. For religious stimuli, there were additional regions of the brain that did differ by group, however these results seem best explained by a common reaction in both groups to statements that violate religious doctrines (discussed further below).

The opposite contrast, *disbelief minus belief*, yielded increased signal in the superior frontal sulcus and the precentral gyrus. The engagement of these areas is not readily explained on the basis of prior work (see Table 2).

**Table 2:** Disbelief minus belief.

Brain region	hemi	x	y	z	Peak Z score
Postcentral gyrus	L	−44	−24	54	4.41
Superior frontal sulcus	R	24	14	48	3.36

### Religious compared with Nonreligious statements

**Figure 2:**
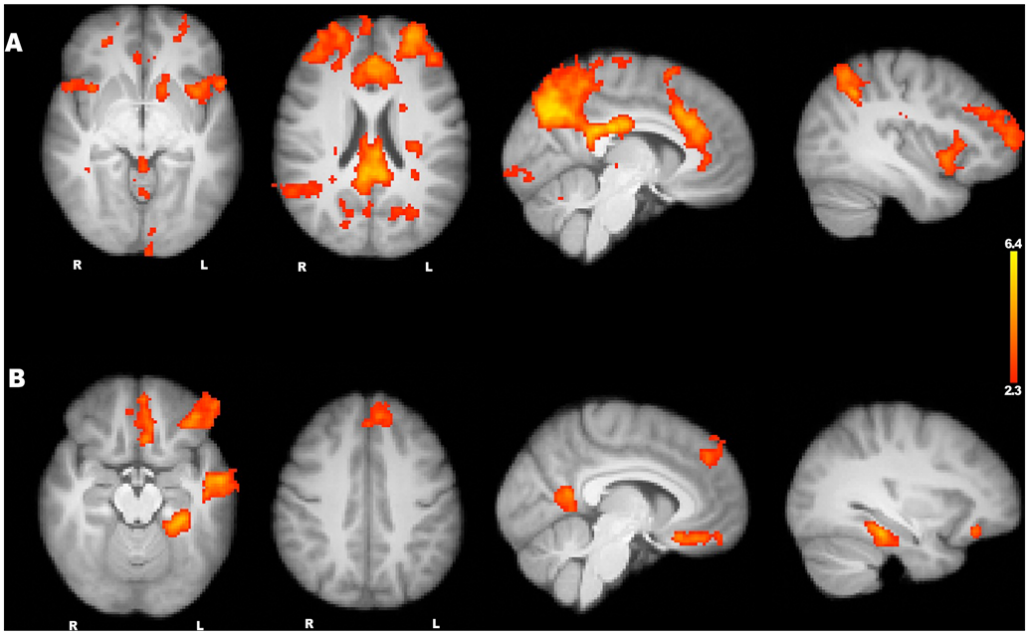
Religious versus nonreligious statements. (A) The MRI signal was greater when subjects evaluated religious statements compared with nonreligious statements in areas throughout the brain, including the precuneus, anterior cingulate, insula, and ventral striatum. (B) Increased signal was found for nonreligious statements compared with religious statements in several left hemisphere regions including the parahippocampal gyrus, retrosplenial cortex, temporal pole, middle temporal gyrus and hippocampus.

While the contrast of *belief minus disbelief* yielded similar activation patterns for both stimulus categories, a comparison of all religious trials to all nonreligious trials produced a wide range of signal differences throughout the brain. The contrast of *religious stimuli minus nonreligious stimuli* (see Fig. 2A, Table 3.) revealed greater signal in many regions, including the anterior insula and the ventral striatum. The anterior insula has been regularly linked to pain perception [34] and even to the perception of pain in others [35]. This region is also widely believed to mediate negatively valenced feelings like disgust [36], [37]. The ventral striatum is also regularly associated with emotional processing, especially with reward [38] and appears to play a role in cognitive planning [39]. We also found greater signal for religious stimuli in the anterior cingulate cortex (ACC). The ACC is often taken to be a reporter of response conflict [40], and activity in this region has been negatively correlated with religious conviction [41].

**Table 3:** Religious minus nonreligious statements.

Brain region	hemi	x	y	z	Peak Z score
Posterior cingulate		−2	−22	30	6.27
Precuneus	L	−10	−72	36	6.38
Anterior cingulate		0	30	26	5.09
Frontal pole	L	−32	56	8	5.3
	R	30	60	10	4.78
Anterior insula	L	−36	10	−4	4.09
	R	34	12	−8	3.59
Middle frontal gyrus	R	42	40	26	4.14
Lateral occipital gyrus	L	−32	−62	48	5.03
	R	32	−54	38	3.93
Intraparietal sulcus	L	−32	−56	40	5.09
	R	32	−54	38	3.93
Ventral striatum	L	−16	20	0	3.3
	L	−14	12	−6	3.53
Inferior frontal gyrus	L	−50	10	2	3.9
Superior frontal gyrus	R	12	16	64	4.38
Thalamus		2	−24	6	3.42
Cerebellum		2	−72	−12	3.23

Another key region that appears to be preferentially engaged by religious thinking is the posterior medial cortex. This area is part of the previously described resting state network that shows greater activity during both rest and self-referential tasks [3].

The opposite contrast, *nonreligious minus religious statements*, produced greater signal in left hemisphere networks, including the hippocampus, the parahippocampal gyrus, middle temporal gyrus, temporal pole, and retrosplenial cortex (see Fig. 2B, Table 4). It is well known that the hippocampus and the parahippocampal gyrus are involved in memory retrieval [42]. The anterior temporal lobe is also engaged by semantic memory tasks [43], and the retrosplenial cortex displays especially strong, reciprocal connectivity with structures in the medial temporal lobe [44].

**Table 4:** Nonreligious minus Religious statements.

Brain region	hemi	x	y	z	Peak Z score
Ventromedial prefrontal cortex	L	−4	22	−18	4.1
Superior frontal gyrus	L	−20	34	52	3.54
Middle temporal gyrus	L	−56	−6	−16	4.52
Parahippocampal gyrus	L	−26	−40	−14	5.16
Retrosplenial cortex	L	−14	−52	4	4.62
Orbital frontal	L	−40	38	−16	4.35
Temporal pole	L	−48	16	−34	3.59
Hippocampus	L	−22	−10	−22	3.39

**Figure 3:**
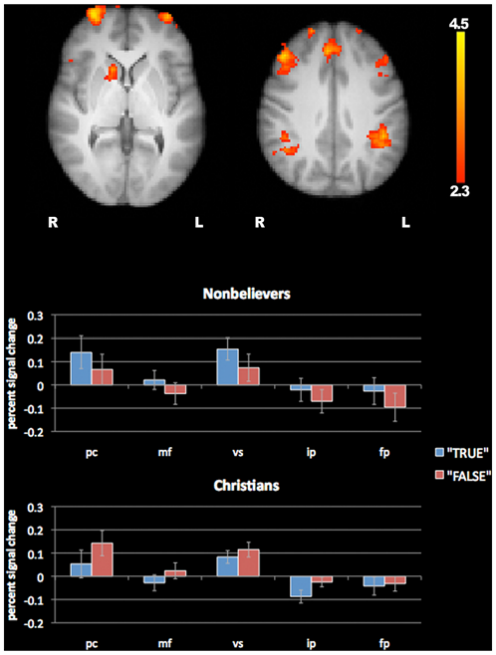
Reponses to blasphemy in both groups. There were significant differences between blasphemous and non-blasphemous statements in both groups. These are regions that show greater signal both when Christians reject stimuli contrary to their doctrine (e.g. “The Biblical god is a myth”) and when nonbelievers affirm their belief in those same statements (pc = paracingulate gyrus; mf = middle frontal gyrus; vs = ventral striatum; ip = inferior parietal lobe; fp = frontal pole). Error bars represent standard error of the mean.

Finally, among our religious stimuli, the subset of statements that ran counter to Christian doctrine yielded greater signal for both groups in several brain regions, including the ventral striatum, paracingulate cortex, middle frontal gyrus, the frontal poles, and inferior parietal cortex (see Fig 3, Table 5). These regions showed greater signal both when Christians rejected stimuli contrary to their doctrine (e.g. “The Biblical god is a myth”) and when nonbelievers affirmed the truth of those same statements. In other words, these brain areas responded preferentially to “blasphemous” statements in both subject groups. This contrast is the result of a double subtraction on religious trials: (Nonbeliever True−Nonbeliever False)−(Christian True−Christian False) = NT−NF−CT+CF = NT+CF−NF−CT = (NT+CF)−(NF + CT). The opposite contrast: (NF−NT)−(CF−CT) produced a null result.

**Table 5:** Double subtraction (“blasphemy” contrast).

Brain region	hemi	x	y	z	Peak Z score
Paracingulate gyrus	R	2	40	34	3.5
Ventral striatum	R	14	16	0	3.52
	R	16	14	−8	3.61
Middle frontal gyrus	R	46	30	34	4.32
	L	−48	36	22	3.07
Frontal pole	L	−36	64	2	3.77
	R	32	64	4	4.5
Inferior parietal lobe	L	−42	−48	46	3.58
	R	48	−48	46	3.39

## Discussion

Nearly a century of opinion polling attests that 70–85 percent of Americans profess not merely a belief in a generic God, but a belief in highly specific, religious propositions: that the Bible is the word of God (whether literal or “inspired”), that Jesus Christ will physically return to earth at some point in the future, that Satan exists and leads people to sin, that prayers actually get answered, etc. The failure to subject such beliefs to rational criticism may be one reason for their survival. But, as Boyer [31], [45] points out, the failure of reality testing cannot explain the specific character of religious beliefs. According to Boyer, religious beliefs and concepts must arise from mental categories and cognitive propensities that predate religion—and these underlying structures might determine the stereotypical form that religious beliefs and practices take. These categories relate to things like intentional agents, animacy, social exchange, moral intuitions, natural hazards, and ways of understanding human misfortune. On Boyer's account, people do not accept implausible religious doctrines because they have relaxed their standards of rationality; they relax their standards of rationality because certain doctrines fit their “inference machinery” in such a way as to seem credible. And what most religious propositions may lack in plausibility they make up for in the degree to which they are memorable, emotionally salient, and socially consequential; all of these properties are a product of our underlying cognitive architecture, and most of this architecture is not consciously accessible. Boyer argues, therefore, that explicit theologies and consciously held beliefs are not a reliable indicator of the contents or causes of a person's religious outlook.

Boyer may be correct in saying that we have cognitive templates for religious ideas that run deeper than culture (in the same way that we appear to have deep, abstract concepts like “animal” and “tool”). We may, in fact, be what Bloom [46] has called “common sense dualists”—that is, we may be constitutionally inclined to see mind as distinct from body and, therefore, will tend to intuit the existence of disembodied minds at work in the world. This could lead us to presume ongoing relationships with dead friends and relatives, to anticipate our own survival of death, and to generally conceive of people as having immaterial souls.

A variety of experiments suggest that children are predisposed to assume both design and intention behind natural events—leaving many psychologists and anthropologists to believe that children, left entirely to their own devices, would invent some conception of God [47]. The psychologist Margaret Evans has found that children between the ages of eight and ten, whatever their upbringing, are consistently more inclined to give a Creationist account of the natural world than their parents are [48].

Because our minds have evolved to detect patterns in the world, we may tend to detect patterns that aren't actually there—ranging from faces in the clouds to a divine hand in the workings of Nature. Hood [49] posits an additional cognitive schema that he calls “supersense”—a tendency to infer hidden forces in the world, working for good or for ill. On his account, supersense generates beliefs in the supernatural (religious and otherwise) all on its own, and such beliefs are thereafter modulated, rather than instilled, by culture. Hood likens our susceptibility to religious ideas to our propensity to develop phobias for evolutionarily relevant threats (like snakes and spiders) rather than for things that are far more likely to kill us (like automobiles and electrical sockets). Barrett [50] makes the same case, likening religion to language acquisition: we come into this world cognitively prepared for language; our culture and upbringing merely dictate which languages we will be exposed to.

And yet, however predisposed the human mind may be to harboring religious beliefs, it remains a fact that each new generation receives a religious worldview, at least in part, in the form of linguistic propositions—far more so in some societies than in others. Whatever the evolutionary underpinnings of religion, it seems unlikely that there is a genetic explanation for the why the French, Swedes, and Japanese tend not to believe in the God of Abraham while Americans, Saudis, and Somalis do. The importance of religious doctrines that purport to be true, and their subsequent acceptance as true by great numbers of human beings, seems indisputable.

Recent attempts to study the neural correlates of religious belief have either suffered from a lack of a nonreligious control condition [2] or were not designed to isolate the variable of belief at all [41]. To investigate the neural correlates of belief for both religious and nonreligious modes of thought, we asked Christians and nonbelievers to evaluate statements of both types while in the MRI scanner.

The data reported above present statistical tests of the reliability of signal changes occurring throughout the brain as a function of the stimuli and their associated behavioral responses. However, these data are of greater value when interpreted against related results in the neuroscientific literature. Such a discussion necessarily entails “reverse inference” of a sort often considered problematic in the field of neuroimaging [51]. One cannot reliably infer the presence of a mental state on the basis of brain data alone, unless the brain regions in question are known to be truly selective for a single state of mind. As the brain is an evolved organ, with higher order states emerging from lower order mechanisms, very few of its regions are so selective as to fully justify inferences of this kind. Nevertheless, our results appear to make at least provisional sense of the emotional tone of belief. And whatever larger role our regions of interest play in human cognition and behavior, they appear to respond similarly to putative statements of fact, irrespective of content, in the brains of both religious believers and nonbelievers.

The contrast, *belief minus disbelief*, revealed greater BOLD signal in the VMPFC (see Fig. 1, Table 1). The medial prefrontal cortex is known to have a high level of resting state activity and to show reduced activity compared to baseline for a wide variety of cognitive tasks [52]. BOLD signal in this region has often been associated with self-representation, particularly for verbal stimuli [3]: for instance, one sees smaller decreases in activity from baseline when subjects make judgments about themselves than when they make judgments about others [53]. This region has also been implicated in reward-related processing [54]. The smaller decrease in activity for belief compared to disbelief could reflect the greater self-relevance and/or reward value of true statements.

Our study was designed to produce high concordance on nonreligious stimuli (*e.g.*, “Eagles really exist”) and high discordance on religious stimuli (*e.g.*, “Angels really exist”). The fact that we found essentially the same signal maps for *belief minus disbelief* in both groups, on both categories of content, argues strongly for the content-independence of belief and disbelief as cognitive processes. Despite the fact that religious believers and nonbelievers accepted and rejected diametrically opposite statements in half of our experimental trials, the same neural systems were engaged in both groups throughout. This would seem to rule out the possibility that these results could be explained by any property of the stimuli apart from their being deemed “true” or “false” by the subjects in our study. The involvement of the VMPFC for belief is consistent with our earlier findings [1].

In our earlier study of belief, we found anterior insula signal to be associated with the contrast *disbelief minus belief*. Kapogiannis et al. [2] also found signal in the insula to be correlated with the rejection of religious statements deemed false. The significance of the anterior insula for negative affect/appraisal has been discussed above. Because Kapogiannis et al. did not include a nonreligious control condition in their experiment, they interpreted the insula's recruitment as a sign that violations of religious doctrine might provoke “aversion, guilt, or fear of loss” in people of faith. Reducing the statistical thresholding in our present study did nominate the insula as a region of interest for disbelief, in both groups and on both categories of stimuli. However, these areas of signal did not survive our cluster thresholding.

Our previous study of belief, in which we explicitly modeled uncertainty, revealed greater signal in the ACC and adjacent regions of the superior frontal gyrus in the uncertainty condition. Given that our signal maps in the contrast *religious minus nonreligious* elicited this same pattern, we speculate that both groups experienced greater cognitive conflict and uncertainty while evaluating religious statements. In support of this conjecture, we also note that our religious stimuli, while semantically and grammatically well matched to our nonreligious stimuli, incurred longer response times for both groups. This contrast also showed bilateral signal in the striatum and the anterior insulae. It is perhaps not surprising that the evaluation of religious statements would more fully engage regions of the brain responsive to emotional salience, both positive and negative.

The contrast *religious minus nonreligious* also showed increased signal in the medial parietal regions regularly associated with self-referential tasks. We note that a possible difference between responding to our religious and nonreligious stimuli is that, for both groups, a person's answers could serve to affirm his or her identity: i.e. for every religious trial, Christians were explicitly affirming their religious worldview, while nonbelievers were explicitly denying the truth-claims of religion.

The opposite contrast, *nonreligious minus religious*, showed increased signal in left hemisphere memory networks. Thus, judgments about the nonreligious stimuli presented in our study seemed more dependent upon those brain systems involved in accessing stored knowledge.

Finally, there were several regions that showed greater signal in both groups in response to “blasphemous” statements (i.e. those that ran counter to Christian doctrine). The ventral striatum signal in this contrast suggests that decisions about these stimuli may have been more rewarding for both groups: Nonbelievers may take special pleasure in making assertions that explicitly negate religious doctrine, while Christians may enjoy rejecting such statements as false.

There is, of course, no reason to expect that any regions of the human brain are dedicated solely to belief and disbelief. Nevertheless, our work suggests that these opposing states of cognition can be discriminated by functional neuroimaging and are intimately tied to networks involved in self-representation and reward. Despite vast differences in the underlying processing responsible for religious and nonreligious modes of thought, the distinction between believing and disbelieving a proposition appears to transcend content. These results may have many areas of application—ranging from the neuropsychology of religion, to the use of “belief-detection” as a surrogate for “lie-detection,” to understanding how the practice of science itself, and truth-claims generally, emerge from the biology of the human brain.

## Materials and Methods

### Experimental Subjects

We enrolled 54 subjects who were (1) between the ages of 18–30, (2) not taking anti-depressants, (3) neurologically healthy, (4) free of obvious psychiatric illness or suicidal ideation, and (5) native speakers of English as their first language. These inclusion/exclusion criteria sought to remove confounding effects of (1&2) age- or drug-related hypometabolism in the brain, (3) structural and functional anomalies due to illness or injury, (4) differences in psychological health, and (5) differences in linguistic processing. Subjects with implanted metal are routinely excluded from experiments using magnetic resonance imaging (MRI) for reasons of safety. All subjects gave written, informed consent according to the guidelines of the UCLA Human Subjects Protection Committee.

In order to implement these inclusion/exclusion criteria, subjects were screened by means of a telephone questionnaire. This questionnaire allowed us to isolate the variable of religious belief, in an effort to admit only dedicated Christians and nonbelievers into the protocol.

Once we had two groups of subjects (Christians and Nonbelievers), we attempted to balance these groups with respect to 1) general reasoning ability, 2) age, and 3) years of education. We also sought to exclude all subjects who exhibited signs of psychopathology. To this end we assessed subjects' general intelligence using the Weschler Abbreviated Scale of Intelligence (WASI) and screened for psychopathology using the Brief Psychiatric Rating Scale (BPRS). Subjects were not given the results of these tests.

Thirteen subjects were excluded on the basis of these psychological assessments. This left us with 41 subjects (19 female, 22 male; 20 Christians; 21 Nonbelievers). Forty of these participated in the fMRI portion of our study, but ten were later dropped, and their data excluded from subsequent analysis, due to technical difficulties with their scans (2 subjects), or to achieve a gender balance between the two groups (1 subject), or because their responses to our experimental stimuli indicated that they did not actually meet the criteria for inclusion in our study as either nonbelievers or committed Christians (7 subjects).

While gradations of belief are certainly worth investigating, our experiment sought to characterize belief and disbelief in their purest form. It was, therefore, essential that we exclude subjects who could not consistently respond “true” or “false” with conviction. Our decision to exclude data from subjects whose answers were not consistent with our pre-screening criteria was part of our original design and was not made based on any evaluation of the scanning data (the fMRI data from these subjects were never analyzed). While we adopted the criteria of excluding anyone who responded to one category of statements with less than 90% predictability, the 7 subjects who were excluded on this basis had responses that ranged from 22% to 43% discord with the expected responses. (For instance, one subject who passed our initial screening as a nonbeliever actually agreed with 43% of the religious Christian statements once inside the scanner.) Because our telephone questionnaire needed to screen for all relevant variables (age, native language, MRI safety issues, etc.), it contained only a very abbreviated assessment of belief. Thus, the high exclusion rate at this later stage of the experiment represents the failure of our brief screening procedure to accurately assess a person's religious beliefs, rather than a bias in our approach to data analysis. These exclusions ensured that our final group of subjects did, in fact, strongly believe/disbelieve our religious stimuli. We note, however, that the subjects retained in this experiment do not represent the full range of religious commitment found in the general population.

Our final study consisted of data acquired from 30 subjects (15 Christians; 15 Nonbelievers; 7 men and 8 women in each group). The mean full-scale WASI scores, years of education, and ages for the groups appear in Table 6.

**Table 6:** Subject Data: The mean full-scale WASI scores, years of education, and ages for all subjects retained in this experiment.

GROUP	WASI	EDUCATION	AGE
**Christians (all):**	125.6	15.1	22.0
**Nonbelievers (all):**	124.7	15.1	21.6
**Christians (male):**	127.6	15.3	22.7
**Christian (female):**	123.9	14.9	21.4
**Nonbelievers (male):**	123.7	14.6	21.3
**Nonbelievers (female):**	125.5	15.6	21.9

### Experimental design

Once inside the scanner, subjects were presented with a series of short statements through a video-goggle display (Resonance Technology, Inc). After reading each statement, they were asked to evaluate its truth content with the press of a button, indicating “true” (belief), “false” (disbelief), and “undecidable” (uncertainty). The presentation of stimuli was self-paced. Stimuli were drawn from two categories, religious and nonreligious. All statements were designed to be judged easily as “true” or “false” (the response of “undecidable,” while available to subjects, was not expected).

Within each category, we attempted to balance the stimuli with respect to semantic structure and content. Strict balancing across categories was not possible, however, as the two categories differ with respect to content, in principle. For the purposes of stimulus design (not presentation) we generated our statements in groups of four (true and false; religious and nonreligious):

**The Biblical God really exists.** (Christian true/nonbeliever false)

**The Biblical God is a myth.** (Christian false/nonbeliever true)

**Santa Claus is a myth.** (Both groups true)

**Santa Claus really exists.** (Both groups false)

Christians and Nonbelievers were expected to respond identically to nonreligious stimuli and to be discordant for all religious trials. The nature of the questions, along with a telephone screening protocol that selected for nonbelievers and committed Christians, more or less ensured that subjects' responses would segregate in this way (see Supplementary Information: Experimental Stimuli S1).

Prior to scanning, all stimuli were tested to ensure that they would function appropriately in our experiment. For this purpose, we created several sets of candidate stimuli and solicited responses from the nonbelievers and Christians on the Internet. For each statement the number of respondents averaged around 5000, 80–90% of whom were nonbelievers. The numbers of committed Christians responding to each statement ranged from 254–787. Participants were asked to judge the veracity of each statement using a Likert scale (ranging from 1-“strongly disbelieve” to 5-“strongly believe”). In selecting stimuli for this study, we retained only those statements that reliably elicited ratings of 1 or 5 in these surveys. We kept only those religious statements that segregated along the lines of stated belief (Christian v. nonbeliever), and only those nonreligious statements that showed no such interaction.

Each functional scan was balanced with respect to category content (religious/nonreligous) and response valence (true/false). After scanning, subjects were asked to review their recorded responses to all statements to ensure that they reflected their actual beliefs at the time of scanning. Erroneous responses, responses of “undecided,” or those statements which, upon debriefing, could not be clearly judged by subjects to be “true” or “false” were excluded from subsequent data analysis.

The stimuli were presented in an order optimized to produce maximal signal differentiation and to ensure temporal jitter between trials using a genetic optimization algorithm [55]. Jitter was achieved by interspersing the task trials with fixation trials in an order determined by the genetic algorithm. The presentation of each of three stimulus sets was randomized for each subject. For the purposes of data analysis, an experimental trial began the moment a statement appeared and ended with each subject's response.

### Functional MRI Data Acquisition

All scanning was performed on a Siemens Trio 3T scanner. Each subject received three functional scans of approximately 6 to 10 minutes in length. Functional images were acquired in the AC-PC orientation using T2*-weighted echo-planar scans (TR = 2000 ms, TE = 35 ms, flip angle = 80 degrees, FOV = 192×192 mm, slice thickness = 3 mm, number of slices = 29, inter-slice gap = 1 mm, bandwidth = 3256 Hz/pixel). FMRI data processing was carried out using FEAT (FMRI Expert Analysis Tool) Version 5.98, part of FSL (FMRIB's Software Library, www.fmrib.ox.ac.uk/fsl). Registration to high resolution structural and to standard space images was carried out using FLIRT [56], [57], [58]. We used FLIRT to register the functional data to the atlas space in three stages. First, functional images were aligned with the high-resolution co-planar T2-weighted image (TR = 5000 ms, TE = 31 ms, flip angle = 90 degrees, FOV = 200×200 mm, slice thickness = 3 mm, slices = 29, inter-slice gap = 1 mm, bandwidth = 1628) using 6 degrees of freedom rigid-body warping procedure. Next, the co-planar volume was registered to the T1-weighted MP-RAGE (TR = 1900 ms, TE = 3.43 ms, TI = 900 ms, flip angle = 9 degrees, FOV = 256×256 mm, slice thickness = 1 mm, number of slices = 160, inter-slice gap = .5 mm, bandwidth = 180 Hz/pixel) using a six degrees of freedom rigid-body warp. Finally, the MP-RAGE was registered to the standard MNI atlas with a twelve degrees of freedom affine transformation. Registration from high resolution structural to standard space was then further refined using FNIRT nonlinear registration [59], [60].

### Functional MRI Data Analysis

All functional data were analyzed using FSL. We performed standard preprocessing—slice timing correction, motion correction, brain extraction, spatial smoothing (using a 5 mm kernel), high-pass filtering, and pre-whitening—prior to contrast modeling. Individual responses were analyzed in an event-related manner. We modeled four types of trials with separate regressors: nonreligious true, nonreligious false, religious true, and religious false. Since response time varied among conditions, we also included in our model an additional regressor to account for the effects of response time. This regressor had a height equal to the response time for each trial, and was orthogonalized with respect to the other four regressors. The six motion correction parameters were also included as additional regressors. Our maps of blood oxygen level dependant (BOLD) signal changes were the result of pairwise contrasts between each of the task conditions. Statistical images were thresholded using clusters determined by Z >2.3 and a corrected cluster size significance threshold of p = 0.05.

## Supporting Information

Experimental Stimuli S1 The full set of stimuli used in this experiment.
(0.05 MB DOC)

Table S1 (0.04 MB DOC)

Figure S1 (0.08 MB TIF)
