# Correction: The Neural Correlates of Religious and Nonreligious Belief

**DOI:** 10.1371/annotation/7f0b174d-ab93-4844-8305-1de22836aab8

**Published:** 2010-01-14

**Authors:** Sam Harris, Jonas T. Kaplan, Ashley Curiel, Susan Y. Bookheimer, Marco Iacoboni, Mark S. Cohen

The Funding section included some information that relates to competing interests. The Competing Interests section should read: Sam Harris, one of the first authors on this study, is also a co-founder and CEO of The Reason Project. The Reason Project is a nonprofit foundation devoted to the spread of scientific thought and secular values in society. This affiliation does not alter the authors' adherence to all PLoS ONE policies on the sharing of data for the purposes of academic, noncommercial research. 

