# Supplementary material for: The Neural Correlates of Religious and Nonreligious Belief
Source: PLoS One. 2009 Oct 1;4(10):e7272. doi: 10.1371/journal.pone.0007272 (PMC2748718; doi:10.1371/journal.pone.0007272)
Supplement: Experimental Stimuli S1 — The full set of stimuli used in this experiment. (0.05 MB DOC) [file pone.0007272.s001.doc]

Experimental Stimuli S1

Note: Our stimuli were presented to subjects in pseudorandom order. Here we present in ordered quadruples to give an indication of how they were balanced.

Christian True (Atheist False)

Christian False (Atheist True)

Factual True

Factual False

1.

The Biblical God really exists.

The Biblical God is a myth.

Superman is a fictional character.

Superman really exists in the world.

2.

The Bible is free from error.

The Bible is full of fictional stories and contains historical errors.

Some books are full of errors and misconceptions.

All books provided perfectly accurate accounts of history.

3.

People who believe in the biblical God often do so on very good evidence.

People who believe in the biblical God do so on bad evidence.

People who believe in voodoo do so on bad evidence.

People who believe in voodoo do so on very good evidence.

4.

People should be confident that God actually hears their prayers.

People who think they are communicating with God are probably wrong.

People often talk to one another on the telephone.

People now generally communicate by Morse Code.

5.

Christian teaching represents humankind’s most important source of truth.

Christianity does not offer the greatest insight into the human condition.

Science has produced some useful technologies.

Science has never discovered anything new or useful.

6.

It reasonable to believe in an omniscient God.

It is unreasonable to believe in an omniscient God.

It is unreasonable to believe that the earth is flat.

It is reasonable to believe that the earth rotates on its axis.

7.

The Bible is the most important book we have.

The Bible is a highly imperfect book.

The U.S. Constitution is a very famous document.

The U.S. Constitution was first written in Chinese.

8.

Jesus Christ will literally save all human beings who accept his grace.

Jesus Christ can’t do anything to help humanity in the 21^st^ century.

If the U.S. economy fails, many other countries will feel the effect.

The United States is one of the poorest countries on earth.

9.

It is important to raise children with a belief in God.

It is misleading to tell children that there is a God.

It is important to teach children to read and write.

Children are born knowing how to read and need not be taught.

10

Human beings were created by God.

The Biblical God is a human invention.

The airplane is a human invention.

All airplanes are exactly the same size.

11.

It is a good idea to trust in Biblical teachings.

It is a bad idea to take the teachings of the Bible to be true.

It is generally good to be friendly toward other people.

It is always good to place one’s total trust in a stranger.

12.

Jesus Christ really performed the miracles attributed to him in the Bible.

The miracles described in the Bible are almost certainly fictional.

Alexander the Great was a very famous military leader.

Alexander the Great was probably able to fly and wield other magic powers.

13.

Believing in the Christian God is important for true happiness.

Believing in the Christian God is not important for true happiness.

Eating the wrong foods can negatively affect a person’s health.

It is impossible to eat too large a quantity of healthy food.

14.

Some Christians will probably go to heaven after death.

Devout Christians will go the same place everyone else goes after death.

Good people can still experience suffering.

Good people always experience perfect health.

15.

Angels really exist.

Angels are fictional creatures.

Eagles really exist.

Eagles are very common pets.

16.

Jesus was literally born of a virgin.

The virgin birth of Jesus is a myth.

Childbirth can be a painful experience.

Childbirth entails no danger whatsoever.

17.

The Biblical story of creation is basically true.

The Biblical story of creation is purely a myth.

Bill Gates is one of the founders of Microsoft.

Bill Gates probably never used a computer.

18.

A person must follow the will of God to be truly moral.

True morality has no necessary connection to God.

Moral behavior is important for a healthy society.

A high rate of crime is very good for society.

19.

The Bible is the best guide to morality and personal fulfillment that we have.

Most of the Bible is inferior to modern thinking on morality and human happiness.

Popular books sometimes discuss the subject of morality and human happiness.

Popular books never mention the subject of morality and human happiness.

20.

The universe is a manifestation of God’s creative power.

We can understand the universe without any notion of God.

There are events in the universe that we cannot control.

Human beings now have a complete understanding of the universe.

21.

Christian teaching is the most important system of beliefs we have.

Christian teaching is filled with obvious misconceptions about the world.

Engineers are helpful for designing safe buildings.

Engineering is a totally useless profession.

22.

Human beings literally inherit Original Sin.

Original Sin is nothing more than a myth.

A person’s sense of right and wrong can gradually develop throughout his life.

We are all born knowing exactly how to behave in all circumstances.

23.

There is one God, eternally existent in three Persons: Father, Son, and Holy Spirit.

The Christian doctrine of the Trinity is almost surely fictional.

The U.S. Government has three branches: Executive, Legislative, and Judicial.

The U.S. Government has only three employees.

24.

The Holy Spirit exists and intervenes in human affairs.

The Christian idea of the Holy Spirit is likely fictional, misleading, or empty.

It would be extremely useful if we learned to grow food in any environment.

Human beings have complete control over the environment and can grow food anywhere.

25.

Jesus Christ, born of a virgin, is God come in the flesh.

If Jesus existed, he was conceived naturally, like every other human being.

Shakespeare is one of the most famous writers in history.

Shakespeare is never mentioned in college or high school.

26.

Humans are by nature sinful and inherently in need of Christian salvation.

Humans are a product of the natural world, just like all other animals.

Humans share certain general qualities, regardless of culture.

Human cultures are all exactly the same.

27.

Christ's death provides atonement for the sins of humanity.

Christ’s death can do nothing to redeem the rest of humanity.

Many people keep their money in a bank.

Every person on earth now keeps a lot of money in a bank.

28.

Personal salvation comes to humankind by grace through faith.

It is silly to think that grace and faith can lead to personal salvation.

People often disagree about politics.

People never change their political views.

29.

Jesus Christ literally rose from the dead.

Jesus Christ probably did not rise from the dead.

Some people are afraid to fly in airplanes.

Most people are fully trained airline pilots.

30.

Jesus Christ literally will come to earth again in the Second Coming.

Jesus Christ is very unlikely to return to earth in a Second Coming.

Michael Jackson has been a controversial figure in recent years.

Michael Jackson is very likely to become President of the United states.

31.

Satan actually exists as a force of evil in the world.

Satan is almost certainly fictional.

Certain diseases can spread by casual human contact.

All diseases are equally contagious.

32.

All who die truly accepting Christ will spend eternity in heaven.

After death, it will not matter if a person accepted Christ as his savior.

Many people worry about death.

Most people are eager to die.

33.

Marriage should be a sacred union, exclusively between man and woman.

Marriage is a purely human convention and can be defined in different ways.

Marriage is a very common practice throughout a wide variety of cultures.

It is very important to marry someone who shares your exact birthday.

34.

God has absolute sovereign authority over the natural world.

There is no God who holds authority over the natural world.

The universe is more vast than most ancient men and women realized.

The universe has fewer than one thousand objects in it.

35.

Jesus ascended to Heaven and is seated at the right hand of God the Father.

The belief that Jesus ascended to Heaven is clearly false.

Becoming an astronaut requires very rigorous training.

Most people attempt to be astronauts at some point in their lives.

36.

The Bible contains accurate prophecies that we should take seriously.

Belief in Biblical “prophecy” is based on poor scholarship, wishful-thinking, or both.

Economists often try to predict where the economy is headed.

Economists are now able predict our financial future with perfect accuracy.

37.

Parents should teach their children to value Christianity as a path to truth.

Parents should encourage their children to view all religions as forms of mythology.

Parents should enable their children to keep learning for a lifetime.

Parents should teach their children to avoid all physical exercise.

38.

It is good for political leaders to be believing Christians.

It is good for political leaders to be free of religious beliefs.

It is good for doctors to keep up with changes in medical practice.

It is good for doctors to avoid all direct communication with their patients.

39.

It is important to spread Christianity throughout the world.

Attempting to spread Christianity often causes unnecessary suffering.

It would be good to spread peaceful scientific knowledge throughout the world.

It is important to spread astrology and palmistry throughout the world.

40.

Christianity represents the true path to salvation.

Christianity spreads false ideas about reality.

Some universities are better than others and attract more qualified students.

All universities are equivalent and each attracts the same caliber of student.

41.

The Bible is free from significant error.

The Bible is likely full of errors.

Most magazines make an effort to remove typos from their texts.

There has never been a typo in any professionally printed magazine.

42.

Sin came into the world with the fall of Adam in the Garden of Eden.

The human capacity for evil has nothing to do with the fall of Adam in Garden of Eden.

It can be very useful to speak a foreign language fluently.

Most people in the United States speak at least twelve foreign languages.

43.

The Bible presents an accurate history of the ancient world.

The Bible is a very unreliable record of ancient history.

The ruins of ancient Rome are older than most buildings in America.

Most American cities have buildings that are at least four thousand years old.

44.

It is always best to do one’s work for the glory of God.

The greatest human accomplishments have had nothing to do with God.

The greatest athletes in certain sports are in extremely good physical condition.

All sports require the same degree of physical fitness from their participants.

45.

The most profound truths are to be found in the Bible.

Most profound truths are not in the Bible.

The weather report on the evening news can be very useful.

We can now predict the weather perfectly, anywhere on earth.

46.

Jesus Christ was sent by his Father as a sacrifice for the redemption of humankind.

Jesus Christ was born like an ordinary person, not sent by God.

The Beatles are one of the most famous bands in history.

Very few people have heard the music of the Beatles in the United States.

47.

Humans fell from a blessed state when they yielded to the temptation of Satan.

Humans emerged through a gradual process of evolution like every other species.

Most televisions in the United States have more than one channel.

Most television channels broadcast a continuous series of music videos.

48

God is present in my life.

There is no sign of God in my life.

I generally eat and drink at least twice each day.

I often go weeks without eating or drinking.

49.

People are sometimes correct in thinking that they feel God’s presence.

Anyone who thinks that he can feel God’s presence is mistaken.

The feeling of cool water on a hot day can be quite pleasant.

The feeling of extreme itching is generally pleasant.

50.

God sometimes guides my decisions in life.

God does not influence my decisions in life.

I sometimes seek the advice of my friends and family.

I never speak with my friends or family.

52.

Sometimes I am aware of God’s presence in my life.

I do not experience God’s presence in my life.

I occasionally hear rumors which turn out to be untrue.

Everything I learned in high school must be true.

53.

God sometimes answers our prayers.

There is no God to answer our prayers.

Some businesses are good at responding to their customers questions and concerns.

Businesses are never worried about losing customers.

54.

The idea of God is an invention of human beings.

The idea of God is much more than a product of the human mind.

The idea of democracy has powerfully affected human life.

The idea of democracy has spread to only two countries.

55.

The universe is governed by natural laws that have nothing to do with God.

The universe and its laws are governed by an all-knowing God.

The universe is filled with many stars.

The universe is smaller than the solar system.

56.

A firm religious faith is the most reliable source of happiness.

Religious faith is not necessary for the deepest happiness.

Good friends can be a source of happiness.

Sleep deprivation is a good source of happiness.

57.

Prayer can solve certain problems in the world.

Prayer cannot solve problems in the real world.

Human cooperation is useful for solving problems in the world.

Human cooperation is best established by lying.

58.

God will eventually reward those who live the true faith.

God does not reward anybody for anything.

Some people find volunteer work very rewarding.

All people find dangerous sports rewarding.

59.

The gift of immortality has been revealed by God in the Bible.

The gift of immortality promised in the Bible is unlikely to be real.

Giving gifts to friends can be a source of joy.

Giving gifts is never appropriate between family members.

60.

It is wise to believe that a Personal God is in control of the universe.

It is foolish to believe that a Personal God is in control of the universe.

It is foolish to expect a government to make every one of its citizens perfectly happy.

It is wise to create a government that can help protect its citizens from harm.

61.

The existence of God is the best explanation for the beauty of the universe.

The existence of God is an inadequate explanation for the beauty of the universe.

The existence of the mythical fountain of youth has not been established by science.

The existence of the mythical fountain of youth has been established beyond any doubt.
