# Supplementary material for: The Neural Correlates of Religious and Nonreligious Belief
Source: PLoS One. 2009 Oct 1;4(10):e7272. doi: 10.1371/journal.pone.0007272 (PMC2748718; doi:10.1371/journal.pone.0007272)
Supplement: Table S1 — (0.04 MB DOC) [file pone.0007272.s002.doc]

| **SUBJECT** | **ALL** | **Religious** | **Nonreligious** | **""True"** | **""False"** |
| --- | --- | --- | --- | --- | --- |
| AVG | 3.81 | 3.99 | 3.66 | 3.70 | 3.95 |
| STDERR | 0.15 | 0.15 | 0.16 | 0.16 | 0.15 |
| **NONBELIEVERS** | **ALL** | **Religious** | **Nonreligious** | **""True"** | **""False"** |
| AVG | 3.59 | 3.74 | 3.43 | 3.44 | 3.73 |
| STDERR | 0.24 | 0.25 | 0.23 | 0.26 | 0.22 |
| **CHRISTIANS** | **ALL** | **Religious** | **Nonreligious** | **""True"** | **""False"** |
| AVG | 4.04 | 4.24 | 3.89 | 3.96 | 4.17 |
| STDERR | 0.17 | 0.16 | 0.22 | 0.16 | 0.20 |

**Table S1: Reaction Time Data**
