# Supplementary figures and images for: The Neural Correlates of Religious and Nonreligious Belief
Source: PLoS One. 2009 Oct 1;4(10):e7272. doi: 10.1371/journal.pone.0007272 (PMC2748718; doi:10.1371/journal.pone.0007272)

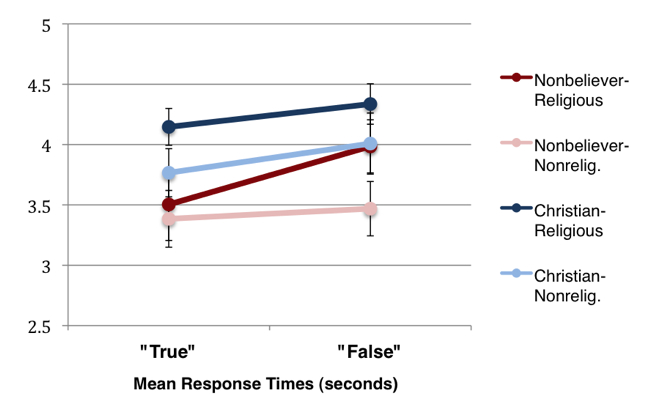

Supplement: Figure S1 — (0.08 MB TIF) [file pone.0007272.s003.tif]
